# The Colonial Clinic in Conflict: Towards a Medical History of the Palestinian Great Revolt, 1936–1939

**DOI:** 10.1007/s11013-022-09779-0

**Published:** 2022-03-22

**Authors:** Chris Sandal-Wilson

**Affiliations:** Present Address: Department of History, University of Exeter, Exeter, UK

**Keywords:** Colonial medicine, Missionary medicine, Medical neutrality, Anti-colonial revolt, Counter-insurgency, Palestine, British mandate Palestine, Hebron

## Abstract

This article reconstructs how Arab doctors, medical missionaries, British counterinsurgents, and Palestinian rebels negotiated and contested the legitimate role of medical workers and healthcare in times of colonial conflict. Drawing insight from a medical anthropological literature which challenges the notion of medical neutrality as normative, and setting mandate Palestine alongside other case studies of medicine in times of conflict from the interwar Middle East and North Africa, this article argues that while healthcare and medical authority could be put to work to support the colonial status quo, they could serve other, more radical ends too. To highlight the complexity of the political positioning of medical workers and healthcare, this article focuses on the town of Hebron during the great revolt which rocked the foundations of British rule in Palestine between 1936 and 1939, and relies on a range of colonial and missionary archival sources. The first part of the article uses the case study of an Egyptian medical doctor who took up political office in the town in moments of crisis to show how medical authority could be consciously transmuted into a force to uphold a besieged political order. The second part draws on the diary of a British mission doctor to reconstruct his efforts to assert medical neutrality during the great revolt, and—more strikingly still—how Palestinian insurgents participated actively in this attempt to transplant international legal protections to Hebron. The final part traces the incorporation of healthcare into the strategies of both British counterinsurgents and Palestinian rebels, with the British policy of collective punishment indirectly but appreciably degrading access to healthcare for Palestinians, and Palestinian counterstate ambitions extending to the establishment of insurgent medical services in the hills.

## Introduction

Between 1936 and 1939, anti-colonial rebellion rocked the foundations of British rule in Palestine. Beginning in April 1936, with a general strike which lasted an unprecedented six months, the great revolt aimed to overturn British rule—formalised in the wake of the First World War as a mandate of the newly created League of Nations—and its commitment to establishing a Jewish national home in Palestine. It quickly morphed into a countrywide armed uprising which was paused in October 1936, resumed in September 1937, and ultimately suppressed in the second half of 1939 as a result of a British counterinsurgency effort which entailed, at its height, the deployment of tens of thousands of British soldiers to Palestine right on the eve of the Second World War. The great revolt is perhaps the most closely studied event in Palestinian history before 1948 (Abboushi 1977; Anderson 2018; Khalidi 2006; Provence 2011; Stein 1990; Swedenburg 2003; Yazbak 2000), while the British counterinsurgency which eventually suppressed it has also been the subject of much historical attention both in its own right (Anderson 2019; Hughes 2009, 2010, 2019; Kelly 2017; Norris 2008), as well as in relation to its prefiguring of the strategies of the Israeli occupation in the Palestinian territories many decades later (Anderson 2019; Khalili 2010). For all the attention the period has received, however, the medical history of the revolt has been overlooked. This article highlights the multiple, profound consequences of the great revolt and its violent suppression for histories of health and medicine in British-ruled Palestine. Focusing on the town of Hebron in the hilly interior of Palestine, it demonstrates that medical workers adopted a range of stances in relation to the ongoing conflict, and argues that for both British counterinsurgents and Palestinian rebels alike, healthcare became yet another terrain of battle.

Introducing a special issue on ‘the clinic in crisis’ in this journal in 2016, Adia Benton and Sa’ed Atshan (2016: 153) argued for the importance of ethnographic accounts in revealing how medical neutrality is negotiated, rather than normative, in times of crisis today. The presumption that the clinic is impartial or safe, they argue, is not borne out by a reality in which these spaces are routinely politicised and embroiled in conflict; medical neutrality must be understood as ‘thoroughly political, social, and cultural’, with doctors, too, ‘always positioned socially and politically’. Taking its cue in part from an observation by Peter Redfield (2016: 263–264), in a response to that special issue, that there is nothing necessarily new in contemporary failures to hold the clinic and its inhabitants apart from conflict, this article insists on the value of historical accounts in deepening our understanding of how medical workers and healthcare services more broadly have been politically and socially positioned in times of crisis. More particularly, attending to these dynamics in the context of European rule in the interwar Middle East underlines continuities between a historic refusal to extend the protections of international law to conflicts involving colonial powers and their non-European subjects, and the continued exclusion of particular sites—Palestine among them—from those international legal norms governing war today (Wilke 2014). Mandate Palestine was certainly not unique in the interwar decades in this respect: whether in Iraq after the First World War (Satia 2006), Syria and Morocco in the 1920s (La Porte 2011; Pedersen 2015), or Ethiopia and Palestine in the 1930s (Pankhurst 1999; Perugini and Gordon 2019; Redfield 2016), neither European powers nor the international system as represented by the League of Nations regarded such conflicts as ‘proper’ wars which needed to be conducted according to international law. But in spite of a flourishing of work on colonial violence, policing, and counterinsurgency (Linstrum 2019; Thomas 2012; Wagner 2016), historians—with notable exceptions (Mahone 2006)—have rarely turned a medical historical lens on these moments of open confrontation between anti-colonial movements and colonial counterinsurgencies, or explored how medical neutrality was negotiated within these contexts.

Though both historic and contemporary parallels can be revealing, there are specific considerations to take into account when reconstructing the political and social positioning of medical workers and healthcare services in the context of mandate Palestine. Although portrayed by both the British and Zionists in Palestine as a senseless eruption of criminal violence (Kelly 2017), the great revolt cannot be understood apart from longer histories of Palestinian economic dispossession, political frustration, and social mobilisation. With the British support for Zionism expressed first in the Balfour Declaration and then enshrined in the text of the mandate for Palestine itself, European Jewish immigration and Zionist land purchasing fuelled a crisis of landlessness among the Palestinian peasantry from the 1920s onwards; at the same time, Palestinian demands for self-determination, democratic rule, and an end to the British commitment to Zionism met with little success (Anderson 2017: 41). By the 1930s, new patterns of collective organising and non-violent action—strikes, boycotts, hunger strikes, civil disobedience—had developed, which increasingly took aim at dislodging the British mandate itself, rather than Zionism alone, and continued alongside the rise of armed insurrection after April 1936 (Anderson 2021). Against this backdrop, it is unsurprising that the Palestinian medical community did not remain outside politics. From 1933 in particular, Palestinian doctors mobilised against what they perceived to be an existential threat in the form of the arrival into Palestine of large numbers of European Jewish doctors (Kozma and Furas 2020: 104–105). And during the great revolt, as well as after, some Palestinian doctors took on highly visible political roles, like the physician and intellectual Dr Tawfiq Canaan, who penned manifestoes about the impact of Zionism on health conditions in Palestine (Nashef 2002: 21–23). This article, by focussing on medical missionaries as well as government-employed doctors in Hebron, de-centres these relatively well-known figures, and draws attention to the full range of positions which medical workers could take across the great revolt—even when they rarely expressed themselves so vocally as Canaan.

Reconstructing the political and social positioning of these medical workers in the absence, often, of more explicit articulations of how they themselves conceived of their relationship to concepts like medical neutrality poses a challenge; similarly, healthcare was seldom directly connected to either rebel or counterinsurgency strategies by contemporary observers or actors. Yet a rich seam of colonial and missionary archival material can nonetheless be used to shed light on both. This article draws on a range of archival sources, including official reports by the health department of the government of Palestine, confidential appraisals of government-employed doctors, correspondence between mandate officials, Anglican clergy, and others, as well as contemporary newspaper accounts. In particular, it makes use of a private diary kept by a British medical doctor, Elliot Forster, who was in charge of an Anglican mission hospital in Hebron, and held clinics in nearby villages, throughout most of the revolt. While Forster’s diary has been used by historians to reconstruct the violence of British counterinsurgency (e.g. Hughes 2009), in this article Forster’s diary is read for what it can reveal about the medical history of this period. It is in part on account of the existence of Forster’s lengthy, revealing diary that this article focuses on Hebron, but there are other reasons too. One of the larger towns in Palestine, with an overwhelmingly Muslim population, and located in the hilly interior of the country where the armed rebels made their base, Hebron was particularly affected by the revolt, right up until its eventual suppression in 1939; more than this, it had been one of the epicentres in a significant uprising in 1929, and, as this article argues, memories of that earlier event shaped the medical history of the great revolt in the town in important ways.

The first part of this article situates the great revolt in Hebron within that longer history, by focussing on one government-employed medical doctor who played an important role both in 1929 and 1936: Dr Ahmed ‘Abd el-‘Al, an Egyptian doctor who served as a medical officer in the mandate’s department of health for more than two decades. While the historiography of colonial medicine has long emphasised the ways in which medical authorities might provide a cover for the dislocations of colonial rule (Packard 1989), formulate scientific discourses legitimising colonialism (Vaughan 1991), and extend the reach of the colonial state through interventions on the body of colonised subjects (Arnold 1993), the story of Dr ‘Abd el-‘Al reveals another facet of this relationship between medical and political authority. In both moments of crisis in 1929 and after 1936, the colonial state sought to transmute ‘Abd el-‘Al’s medical authority directly into political office in order to shore up their hold over the town and area, in a strange echo of an earlier British fantasy from Iraq of uniting the functions of the *hakeem*—the doctor—and the *hakim*—the ruler (Dewachi 2017: 49).

The second part turns to a contrasting biographical case study, this time that of the British missionary doctor Dr Elliot Forster, to open up more fully the question of how medical workers negotiated questions of impartiality and loyalty in times of anti-colonial revolt and colonial counterinsurgency. Forster insisted on treating British police and military personnel, Palestinian civilians, and wounded rebels alike at St Luke’s hospital, Hebron. But as medical anthropologists working on contemporary contexts of conflict have argued, appeals to medical neutrality can often be taken as a political stance *against* authorities and the status quo (Aciksoz 2016; Hamdy and Bayoumi 2016; Redfield 2013). In Forster’s case, his dogged attempt to treat all, regardless of their status in the ongoing conflict, put him on a collision course with local British military authorities, and he was forced, eventually, to abandon this assertion of medical neutrality. But, perhaps more strikingly still, Palestinian rebels were also active participants in this bold but unsteady attempt to transplant international legal norms to Hebron, and so extend those protections across the perceived frontiers of civilisation.

The final part of this article shifts focus to healthcare more widely, to reconstruct how medical services were incorporated into the strategies of Palestinian rebels and British counterinsurgents alike. As historians of other interwar Middle Eastern mandates have shown, experiments in public health and in ensuring colonial order were never far apart, whether in peace or times of crisis (Dewachi 2017: 45–64; Neep 2012: 131–164). A close reading of Forster’s diary, alongside an eclectic range of other archival material, reveals the same to have been true of mandate Palestine. Across the great revolt, the British pursuit of a counterinsurgency strategy of collective punishment degraded provision of and access to medical services in Hebron, even as clinics were for the most part left conspicuously untouched—at least directly. For their part, Palestinian rebels seized the initiative, establishing insurgent medical services of their own in the hills, in line with their wider ‘counterstate’ (Swedenburg 2003) ambitions. Both responses, the article concludes, prefigured in important ways later, more systematic attempts to incorporate medical services into the strategies of the Israeli occupation regime and Palestinian civil society.

## From Medical to Political Authority in Times of Crisis

While certainly the most significant and sustained uprising of the interwar years, the great revolt which began in 1936 was not the only occasion on which Palestinians rose up against British rule and its support for a Jewish national home. Even before the British mandate for Palestine had been confirmed by the League of Nations, there had been rioting against Zionism and the British in Jerusalem in 1920 and in Jaffa in 1921. While the rest of the 1920s were politically quiescent, beneath the surface roiled a deepening crisis of Palestinian landlessness and impoverishment, driven by the twin motor of Zionist land purchasing and a British failure to address agrarian taxation and indebtedness (Anderson 2018: 174–179). With Palestinians pushed to precarious existence at the urban margins by deteriorating conditions in the countryside, and against the backdrop of unrelieved political frustration, clashes over the holy places in Jerusalem sparked widespread revolt in August 1929. In Jerusalem’s Old City, Safad, and Hebron—densely packed urban areas where Jews and Arabs lived in ‘dangerous proximity’ (Pappe 2004: 91)—the uprising took the form of bloody communal violence; in Hebron alone, sixty-seven Jews were killed. That massacre, and evacuation of the remaining Jewish residents of the town over the subsequent decade, has resonated down the decades, taking on symbolic meaning in particular for the Israeli settler movement since 1967 (Campos 2007). Historians have focussed on more immediate legacies, noting how the 1929 uprising prefigured in important ways the great revolt which followed less than a decade later (Sela 1994; Anderson 2018). This section builds on that scholarship by exploring another, striking parallel: the assumption of political authority by a government-employed medical doctor, Dr Ahmed ‘Abd el-‘Al, both in 1929 and then from 1936 onwards.

When violence broke out in the streets of Hebron in August 1929, the town’s medical services were in the midst of their own moment of transition. The British mandate government, in line with its wider policy of devolving responsibility for everyday hospital care to municipalities, missionaries, and other voluntary organisations, had limited itself to operating a small dispensary and casualty post in the town. Hospital provision was left instead to Anglican missionaries, who had been running a small, twenty-bed hospital in Hebron since the 1890s. Although in line with wider government policy, this arrangement left Hebron with notably fewer hospital beds than other towns of a similar size in mandate Palestine. Both Gaza and Nablus had roughly equivalent populations, the vast majority of whom were also Muslim, and while both towns were home to Anglican mission hospitals, these were not only much larger than the one in Hebron, but they also operated alongside municipal hospitals (ARDOH 1929: 44–47). Compounding matters, the mission hospital in Hebron had closed for extensive renovations in July 1928 (ARDOH 1928: 49), leaving the population of the town—around 16,000 in 1929—and the surrounding villages dependent on the government dispensary, a Muslim polyclinic which had only opened in February 1928, and a third clinic run by Hadassah, the American Zionist medical organisation. In May 1929, Dr ‘Abd el-‘Al—the government medical officer for Hebron and the sub-district – wrote to his superiors in Jerusalem to point out that the closure of the Anglican mission hospital had ‘rendered the need for medical relief badly felt in this town’. The new Muslim clinic was ‘not functioning well’, the Jewish clinic was ‘mainly for Jews although treating a limited number of Muslim patients’, and the government dispensary, ‘Abd el-‘Al reported, was overwhelmed and ‘refusing a good number of patients daily reporting for treatment’ as a result.^1^

Medical services were already stretched thin, then, before unrest spread to Hebron late in August 1929. As the department of health later acknowledged, it only had a casualty post with eight beds at its disposal to care for the sixty people wounded in the violence; they ultimately had to be transported to Jerusalem to receive treatment there (Shaw Commission Report 1930: 1032). In spite of the limited resources available to the department of health in Hebron, government medical officer Dr ‘Abd el-‘Al played a key role in the course of events in August 1929. Such was his role, indeed, that his story – ‘Arab doctor saves many’ – was picked up by the *New York Times* (Levy 1929: 7), and he was awarded an honorary O.B.E. in recognition of his actions that year.^2^ In a report submitted to the Royal Commission of Inquiry into the ‘disturbances’, ‘Abd el-‘Al described how he and his tamurgis – medical attendants – joined the small number of police in the town in attempting to restore order and bring the wounded to safety. ‘On more than one occasion’, he recounted, ‘crowds of Jews in hiding, who were met with in the search for the wounded, put themselves under the medical officer’s protection and were escorted to safety’ (Shaw Commission Report 1930: 1032–1033). The *New York Times* had more to say about ‘Abd el-‘Al’s ‘clever strategy’: he had taken large numbers of Jews hiding from the violence and led them to the largest café in Hebron, where many Arab notables were gathered, and placed the Jews under their protection until the unrest had ended. ‘Thus some of the very instigators of the attacks’, the paper observed, ‘found themselves with no alternative but to protect the very persons whom they themselves had given orders to kill’ (Levy 1929: 7). Whether exaggerated or not, the report captures the sense in which ‘Abd el-‘Al appeared to have been able to leverage his authority as a medical doctor to counterbalance other currents in the town’s politics.

The mandate government had made the same observation, apparently, because shortly after the uprising had been quelled, and with the functioning of the municipality severely broken down, ‘Abd el-‘Al was nominated to act as mayor of Hebron temporarily, ‘in order to restore things to normal in town and incidentally make improvements wherever possible’ (Shaw Commission Report 1930: 1033). The value ascribed to ‘Abd el-‘Al’s ability to serve both the mandate’s medical and political interests in Hebron is clear in the confidential annual reports made about him across the subsequent decade. As one appraisal from 1931 put it, he ‘is well liked in his district and has very considerable prestige among the people which is of great value in his work’.^3^ His cachet with the mandate government only increased across the 1930s. Especially following 1933, when large numbers of European Jewish doctors came to Palestine, medicine – as Liat Kozma and Yoni Furas (2020: 101–102) note – became ‘another realm of the Arab–Jewish conflict’, as the Palestinian medical community increasingly organised itself to meet the perceived economic, professional, and nationalist challenge posed by their Jewish counterparts. ‘Abd el-‘Al, who had been born in Egypt, qualified as a medical practitioner in London, and taken up post in the mandate’s health department in 1924, seems to have remained aloof from the wider Palestinian medical community, both politically and socially.^4^

It is unsurprising, then, that when the great revolt began in 1936, the memory of the valuable role ‘Abd el-‘Al had played in restoring order in Hebron in 1929 resurfaced. Elections for a municipal council had been held in 1934, but electoral irregularities, factional rivalries, and the poor health of a polarising mayor, all meant that local colonial officials were already, at the start of 1935, working on ‘finding a more satisfactory successor [to the mayor], capable, respected, and willing to stand for election if required’.^5^ It is not hard to imagine who may have been near the top of their minds. And indeed in September 1936, with the death of the mayor, the murder of the deputy mayor, the loss of a third member of the municipal council, and the resignation of the rest of the councillors as part of the general strike, those same officials requested that a commission be appointed to take over the functions of the municipality. They recommended that Dr ‘Abd el-‘Al be appointed as one of just two members of the commission.^6^ Just as ‘Abd el-‘Al had managed to translate his medical authority into political office in the context of crisis in 1929, across the great revolt the mandate government reported favourably on his value as both medical officer and municipal commissioner. In 1936, government appraisals noted both the services he had rendered to the department of health, as well as how, ‘[d]uring the disturbances his assistance was much appreciated by the military’.^7^ ‘His influence in Hebron and district,’ another report, this time from 1938, read, ‘is considerable and has proved its value during the past troublesome months’.^8^ Even after the revolt had ended, the British continued to rely on him to shore up order in Hebron during the Second World War, re-appointing him as one of the municipal commissioners for the town in October 1940 (Palestine Post 25 October 1940, 2).

The British may have been satisfied with the medical and political service ‘Abd el-‘Al rendered both in 1929 and from 1936 onwards. But it is clear that especially as time wore on, criticism attached itself both to the municipal commission and to ‘Abd el-‘Al personally. In 1938, ‘Abd el-‘Al was abducted and taken before a rebel court, where he was tried – along with a colleague from the department of health – for various, unspecified misdemeanours, which included ‘taking too much money from poor patients’.^9^ If this was a critique of his medical practice, the commission, too, became increasingly unpopular. Mandate officials were swamped with successive waves of petitions from residents of Hebron demanding the termination of the commission and the restoration of municipal elections in the early 1940s, with the commission notably criticised in the summer of 1941 for acting only for its own benefit, and not that of the town.^10^ This may not have been unfounded: as the British district commissioner also noted, confidentially, ‘[a]ffairs in this commission are not too good’.^11^ By the time municipal elections were finally scheduled in 1946, ‘Abd el-‘Al had left Hebron for Nazareth, taking up a new post in the department of health in that town. While it is not implausible that his popularity had been compromised by his overlong involvement with the commission, and that he had left as a result, this does not seem to have been the case. From Nazareth, he continued to play a role in the political and medical life of the town that had been his home for two decades, intervening with the mandate government in support of a new polyclinic in Hebron which would be run by the mayor and funded through voluntary contributions.^12^

The scholarship on colonial medicine has long stressed the ways that medical and political power might intersect and indeed be mutually constitutive. As historian Megan Vaughan (1991, 45) noted, the line between colonial administrator and medical officer could often be blurred in the eyes of colonial subjects, with good reason. The case of Dr Ahmed ‘Abd el-‘Al complicates this in at least two ways. In the first place, it involves far more than merely the perception of an overlap between medical and political spheres; ‘Abd el-‘Al was both medical officer and mayor in 1929, and then medical officer and municipal commissioner for the better part of a decade after 1936. Other medical officers certainly were involved in local and municipal administration in mandate Palestine, but the length of time he combined these roles, and the degree of responsibility conferred on him – as temporary mayor in 1929, and as one half of a two-man commission for the first years of the great revolt – are both striking. And second, ‘Abd el-‘Al was not a British colonial officer, but rather an Egyptian doctor who – like many of his Syrian, Lebanese, Armenian, and Egyptian colleagues in the department of health - lived permanently in Palestine from at least 1924 onwards. Indeed, this section has tracked Dr Ahmed ‘Abd el-‘Al’s biography at such length not only because it provides an insight into the strategies by which the British mandate sought to transmute medical authority into political legitimacy in times of crisis, but because his trajectory is distinctive when set alongside that of many of his peers in this period of increasingly politicised professional organising. ‘Abd el-‘Al, then, highlights the spectrum of positions which could be assumed by Arab doctors in times of conflict and crisis.

## Contesting Medical Neutrality: Medical Missionaries and their Palestinian Colleagues

In August 1929, Hebron’s medical services had struggled to deal with the number of wounded as a result of the temporary closure of the Anglican mission hospital in the town. Less than a month later, however, the hospital was reopened with enlarged capacity under the auspices of the Jerusalem and the East Mission as St Luke’s; Dr ‘Abd el-‘Al, having previously drawn attention to the dangerous gap in provision which had resulted from the closure of the mission hospital for renovations, joined senior Anglican figures in Palestine in inspecting the new building shortly before it opened (Shaw Commission Report 1930: 1032–33). Conspicuous by its absence in 1929, St Luke’s would go on to play a key role in treating the wounded during the great revolt just a few years later. Patients treated at St Luke’s included British police and military personnel, residents of Hebron and the surrounding villages – and, controversially, rebel fighters. While the British mission doctor in charge of St Luke’s, Dr Elliot Forster, doggedly defended providing medical assistance to Palestinian rebels, he came under increasing pressure from British military authorities as a result of this position, particularly in the last year of the revolt. While neither he nor his critics used the term ‘medical neutrality’, this is not in itself striking: in debates about the Geneva Conventions which established that medical personnel in conflict situations should be free to tend, without interference, to the wounded regardless of their allegiance, the value of the term ‘neutrality’ had been deeply contested from the start of the twentieth century (Rubenstein 2021: 35). Drawing on Forster’s private diary and correspondence, this section explores the role played by St Luke’s during the great revolt, and Forster’s—ultimately failed—attempt to assert ‘neutrality’ as a medical missionary in the context of anti-colonial revolt and colonial counter-insurgency.

When St Luke’s reopened in September 1929, it resumed its role not just as Hebron’s one hospital, but as the only hospital within twenty miles. The Anglican bishop in Jerusalem described the newly modernised buildings of the hospital as having received ‘a great welcome from the people of the town’, and as drawing ‘considerable numbers’ of patients from the villages, ‘sometimes walking two or three days to reach the hospital’ (MacInnes 1931: 937–938). After a succession of short-lived appointments to the hospital, Dr Elliot Forster took charge of St Luke’s in 1933, a post he retained until the outbreak of the Second World War when he signed up for service in the Royal Army Medical Corps. But in the spring of 1936, he was seriously ill, such that he was in England on sick leave when the great revolt began. The hospital thus was left under the charge of Dr Joyce MacInnes, the daughter of the late Anglican bishop in Jerusalem, for the initial phase of the great revolt. With Hebron shut down almost entirely for the six-month general strike from April, and with frequent shootings both in the town itself as well as the roads out of it, MacInnes was praised by her superiors in the Anglican church for having ‘carried on gallantly in very trying circumstances’. While attendance at the hospital had dropped – ‘because patients are afraid to come’ – the local strike committee had offered MacInnes a label for the windscreen of her car, in order to protect it from damage while she was going about her rounds in the town.^13^

This protection continued to be extended to St Luke’s in the second phase of the great revolt, when the centre of gravity shifted from strike committees in the towns to rebel fighters in the country. Once he had returned to Palestine late in 1936, Forster resumed his visits to nearby villages, where he ran essential weekly clinics for the villagers in the district. As he reported back to his superiors in April 1938, these visits were on a set timetable, and were thus ‘well known to the gentlemen responsible for the hold-ups and the shootings’. But in spite of this, ‘at no time have we suffered let or hindrance’; even when they made contact with ‘the gang’, they had been allowed to pass ‘without molestation’.^14^ This was of no small significance: the Arabic-language press is full of reports of armed gunmen holding up traffic on the roads to and from Hebron across 1938.^15^ Indeed, Forster’s diaries make clear that this protection extended much further than simply tacit non-interference with his work: in the second half of 1938, local rebel leadership actually provided him with an escort for some of his journeys out of Hebron, so that he could travel safely.^16^ While the rebels assured Forster and his Palestinian colleagues at the hospital that ‘none of the local people would touch the doctor’, the difficulty, they noted, was that there were ‘a good many “tough eggs” from foreign parts whose behaviour could not be guaranteed’.^17^

Against this backdrop of hold-ups and abductions on the road, it is unsurprising that British authorities also voiced concerns about Forster’s frequent journeys throughout the district, and proposed that he either stop these visits entirely or travel with a police escort. In an attempt to ensure distance between St Luke’s and the British civil and military authorities, however, the Anglican bishop in Jerusalem intervened on Forster’s behalf:

‘[H]e goes amongst the villagers as a doctor and they know his errands are those of peace and mercy and I personally should strongly deprecate any escort for him as this might be interpreted either that he was afraid or that he was in touch with the police or that he was a government agent.’^18^

The care taken here not to be too closely associated with British police or military forces can be understood as part of a wider attempt by the Anglican church across the mandate period to position itself as a ‘third party’, between the mandate government and the Palestinian population (Okkenhaug 1999; Småberg 2013).

Beyond simply maintaining distance between St Luke’s and the British military authorities on the ascendance across the second half of the great revolt, Forster was a vocal – and well-connected – critic of the military’s counterinsurgency methods (e.g. Hughes 2009: 339–341). The most notable instance of this came in August 1938, when, following a night-time raid on the town by a sizeable rebel force, the police and military responded by imposing a curfew the next morning which was poorly publicised but ruthlessly enforced. In addition to two dozen men injured, many of them with broken crowns, two men – one of them an elderly deaf man – were shot and killed outright, and a further six Palestinians – two of them old men, three of them children – were brought to Forster with gunshot wounds for treatment. One – a boy of fifteen – later died. Forster sent his ‘personal impressions’, complete with detailed descriptions of the gunshot wounds inflicted and the amputations he had had to perform on two of the wounded, straight to the High Commissioner.^19^ Such private reports never, as Matthew Kelly (2017: 126) notes, gained public traction, not least because of the injunction placed on all Palestinian newspapers against reporting the details of military or police operations unless authorised by the government. Forster’s ‘personal impressions’, as he called them, are nonetheless striking for the way they marshal his first-hand, clinical experience to draw out in graphic detail the consequences of the policing method adopted by the British in Hebron in August 1938. If ‘Abd el-‘Al’s medical authority could be put to work to shore up colonial administration, Forster’s clinical expertise was here deployed to critique the British counterinsurgency regime. Yet in an ironic turn of events, Forster’s ‘unwearying, sympathetic, and gratuitous treatment of the large number of casualties’ was credited with having ‘done much to counteract popular indignation’; the assistant district commissioner went so far as to call him ‘the greatest asset that government has in this town and sub-district’.^20^ While the commissioner shared Forster’s criticisms of the military’s excesses, he nonetheless suggested that Forster’s actions – as a British doctor, in an Anglican mission institution – had helped take the edge off this episode. And indeed, it was the strenuous efforts of Forster, the rest of the staff at St Luke’s, and the other government medical doctors in the town which were highlighted in an initial report on the incident in *al-Sirat,* a Jaffa-based daily paper (22 August 1938: 2).^21^

While Forster’s travels around the countryside to hold village clinics, and his responses to British counterinsurgency tactics, both necessitated a degree of careful – if not always successful – distancing from the military authorities, it was in relation to the question of Palestinian access to treatment at St Luke’s that he sought to assert his neutrality in the most explicit and sustained way. In October 1938, Forster complained that wounded Palestinians were not coming to St Luke’s for treatment, ‘fearing not so much from us as from the police and soldiers who must have access to the hospital and who bring here their own wounded’.^22^ They had good reason to be concerned. In January of that year, a man had been admitted to St Luke’s as an in-patient for treatment of a peritonsillar abscess, and while he was at the hospital, the police arrived with a warrant for his arrest as a member of one of the local rebel leaders’ ‘gang’. A constable was set to guard him in the ward, while they waited for a police vehicle to arrive with which to remove him, but the man simulated violent abdominal pain, and when the other patients all corroborated his story that he had been given a purge – that is, a laxative – the constable allowed him to go to the lavatory. ‘After about half an hour’, Forster recounted, as ‘the policeman was still sitting with his mouth open, someone asked him if he was going to sit there all day’. The next day, the escaped patient arranged for his hospital clothes to be returned, along with the fee due for his treatment, and even a bunch of flowers for Forster as a token of thanks.^23^

While in this instance the man had been able to escape, the episode was part of a wider pattern of police targeting suspected rebels while in hospitals (Hughes 2019, 383). In the most notorious example of this practice, British police entered a private hospital in Jaffa and shot and killed an injured man, Ibrahim ibn Khalil, while he was still lying in his hospital bed, in June 1939. The police subsequently reported that he had been ‘shot while trying to escape’. As it turned out, the police had been seeking another target, the principal witness against a British police sergeant and a Jewish advocate who were to be tried for conspiracy, and had killed the first wounded man they could find in their rush to secure a reward. After the assassination, the director of the hospital, Dr Dajani, had his own house searched, and his family intimidated (Hughes 2019: 327–328). And a British solicitor based in Jaffa, acting as the deceased’s legal representative, later complained he had had ‘a battle’ with the coroner in the case, the district officer, over the shooting: the coroner had refused to grant the solicitor access to the coroner’s inquest proceedings, in spite of the fact that he was representing the deceased.^24^

Although this particular incident was in the future, by 1938 Forster’s own experiences would have made it perfectly clear to him that his hospital and patients were not automatically afforded any special, inviolable status which set them apart from the general conditions of the country at this time of revolt and counterinsurgency. In October 1938, then, he worked to secure that status for his hospital, reaching an agreement with the local British civil and military authorities in Hebron by which all patients at St Luke’s were to be ‘exempt from interrogation without [Forster’s] express permission, to be refused at [his] discretion’.^25^ This agreement, which Forster referred to as ‘our Geneva convention’, became well known to the rebels, at least some of whom appeared to quickly place their trust in its protection. That same month, the man who had escaped from the police while a patient in the hospital earlier that year, was brought to St Luke’s with a bullet wound – precisely the kind of injury which would have marked him out as a probable rebel in the eyes of the authorities, and invited interrogation and detention. While he ultimately succumbed to his wound, ‘[t]he fact that his people brought him here again after this first incident’, Forster remarked, ‘shows that they place confidence in our more recently established Geneva convention’.^26^ Others were a little less trusting: later that year, Forster was asked for ‘a guarantee of good faith in respect of our Geneva convention for the treatment of [rebel] wounded’.^27^ But once one of the staff at the hospital – Khalil Jubrail, who regularly served as Forster’s go-between in communicating with the rebels – declared himself willing to be killed if any harm came to the rebels while at the hospital, wounded rebels were indeed brought to St Luke’s for medical care.

That the inviolability of St Luke’s and its patients had to be explicitly negotiated by Forster is unsurprising. His local ‘Geneva convention’ was necessary in view of the systematic failure to extend the norms of international law – including the ‘actual’ Geneva conventions – to conflicts outside Europe across the interwar years, evident in the French bombing of Damascus in 1925 (Pedersen 2015), the use of chemical weapons by the Spanish in Morocco in the same decade (La Porte 2011), and – beginning just before the great revolt in Palestine – the Italian bombing of Red Cross facilities in Ethiopia (Pankhurst 1999; Perugini and Gordon 2019; Redfield 2016). These were not aberrations: as Adom Getachew (2019: 66–67) has recently argued, Italian war crimes in Ethiopia followed the same logic which had underpinned the unequal integration of Ethiopia into the international community across the previous decade. If ‘the international law of armed conflict was… not’, Christiane Wilke (2014) reminds us, ‘intended to protect colonized peoples from oppression’, historians have nonetheless drawn attention to demands by Syrians, Moroccans, Ethiopians, and others for those norms to be extended. While attention has been given to these lobbying efforts as they reached Geneva and other European capitals, including by Palestinians (Wheatley 2015), the story of Forster’s local ‘Geneva convention’ suggests that Palestinian rebels engaged with the project of extending the protection of these international norms on the ground, too, and not merely as petitioners.

For their part, the British military became increasingly furious at Forster’s medical aid to the rebels. By April 1939, Forster suspected that the local military authorities considered him ‘a centre, if not of sedition, at least of a general resistance to authority’, in part because of his ‘Geneva convention’.^28^ He was later warned by the assistant district commissioner at Hebron that the local battalion were growing ‘more and more dissatisfied with our “Geneva convention”’.^29^ Forster protested – in writing – that the army had never raised their concerns directly with him, continuing: ‘if the military authorities are discontent with our poor little convention, it is surely not too much to hope that they will say so openly, rather than maintaining a strong, silent – is sulky too strong a word? – and unconstructive disapproval.’^30^ Eventually, Forster got what he wanted, and in August 1939 the divisional commander communicated his wish that the convention be ‘indefinitely suspended’.^31^ By this point, as Forster himself confessed, it had become ‘a matter more of principle than of practice’; the hospital had admitted no wounded rebel fighters already for some time before the convention was formally suspended.^32^ Throughout this fraught, largely silent stand-off between Forster and the military authorities in Hebron, what is striking is the extent to which Forster came to perceive his medical neutrality as putting a question mark over his loyalty to his country in the eyes of others. In spite of the fact that the convention had been initially approved by the authorities, Forster found himself having to repeatedly protest his neutrality – ‘I have never given any assistance and comfort to the rebels except of a medical kind’^33^ – and indeed later his loyalty – ‘I do not believe it [i.e. giving medical assistance] to be incompatible with loyalty’^34^ – to his countrymen in Palestine. ‘What sticks in my gills… is the implication that my attitude is disloyal, if not positively dishonourable’, Forster seethed privately in his diary in the summer of 1939.^35^

The guilt experienced by Forster is clearly not on a par with the tremendous risks and pressures from the authorities which doctors in other, contemporary contexts have faced for their work (e.g. Aciksoz 2016: 211–214; Hamdy and Bayoumi 2016: 226), nor did the suspicion which attached to his hospital as a ‘centre of resistance’ lead to the kind of infrastructural violence which Omar Jabary Salamanca (2011) has argued follows from the Israeli resignification of life-sustaining public utilities in Gaza as ‘terrorist infrastructures’ in the early twenty-first century. Yet those Palestinians working at St Luke’s – that is to say, the majority of the staff at the hospital – were more vulnerable to being directly targeted by the military authorities in Hebron as a result of their work.

Khalil Jubrail – Forster’s principal go-between with the rebels – is a case in point. Khalil had been working at the hospital since well before Forster arrived to take charge in the 1930s; in fact, it seems he had been attached to the hospital even before the First World War, serving as the dispenser in the hospital’s pharmacy – in spite of his lack of any formal qualifications.^36^ Although the department of health would issue occasional, half-hearted demands that he sit the assistant pharmacist examinations even into the early 1930s,^37^ his long experience was clearly considered to counterbalance this lack of formal qualifications. In the great revolt, Khalil had enabled Forster to communicate with the rebels, and indeed offered himself up as the guarantee for the ‘Geneva convention’. But in January 1939, he was arrested when his photo – complete with a message of ‘affection and loyalty on the back’ – was discovered in the pocket of a local rebel leader’s coat, seized during a raid; it had been, Khalil explained to a furious Forster, part of his guarantee.^38^ Only Forster’s strenuous lobbying with influential contacts in the civil government prevented him from being sent to Acre central prison.^39^ Across the great revolt, medical workers were forced to negotiate difficult questions around neutrality, complicity, and loyalty. But the fact that Forster’s neutrality, or his ability to position himself as almost a third party in relation to both the rebels and the British, depended to a great extent on Khalil and other Palestinian colleagues risking arrest by acting as his go-betweens, underlines that the room for manoeuvre, as well as stakes, for British doctors like Forster were of a different order of magnitude than those which confronted his Palestinian colleagues.

## Healthcare, Collective Punishment, and Counterstate Formations

In 1940, looking back on the great revolt and its suppression, a report on colonial development and welfare services concluded that ‘of all departments the work of the department of health was least interfered with by the recent disturbances’.^40^ It is clear that this was a relative judgement: across the years of the great revolt, government-employed doctors and nurses were murdered (ARDOH 1936, 12), ambulances and hospital buildings attacked,^41^ and long-awaited and much-needed extensions and improvements repeatedly postponed (ARDOH 1937, 9; 12). Notwithstanding this attempt to understate the impact of the great revolt, the department of health had to concede that Hebron and its sub-district had been an area where services had been more dramatically interrupted by the ‘disturbances’ (ARDOH 1939: 12). The previous sections of this article focussed on two doctors – Dr ‘Abd el-‘Al and Dr Forster – to highlight the very different positions medical workers in one town in Palestine could take up in times of colonial conflict. While keeping its focus on Hebron and the surrounding area, this section zooms out from a biographical approach to consider how healthcare more broadly was not simply affected by but incorporated into the strategies of both British counterinsurgents and Palestinian rebels between 1936 and 1939.

Ill-defined in the department of health’s official reports, the ‘disturbances’ which they credited with disrupting the provision of healthcare in the Hebron area during the great revolt were above all those conditions of insecurity on the roads to and from the town which Forster was only able to navigate with the help of rebel escorts. Even then, Forster himself sometimes had to stay at home, having been warned obliquely by local rebel leadership that ‘the weather was very bad’^42^; at other times, it was the British military authorities who forbade his journeys out to the villages to run his weekly clinics.^43^ In spite of these interruptions, Forster appears to have been able to continue with his work in the villages longer than his counterparts in the department of health. Across the 1930s, two of the most important public health schemes in the Hebron area targeted acute conjunctivitis and endemic – that is, non-venereal – syphilis. The department of health had invested in both schemes on the eve of revolt, assigning two medical officers – Dr Samir Shihab, and Dr Fawzi Khalil ‘Abla – to take charge of these campaigns. Both Shihab and ‘Abla had a more typical educational background than their more senior colleague, Dr ‘Abd el-‘Al, having graduated with medical degrees from the American University of Beirut; they were also seen as notably less reliable by the department, with Shihab, in charge of the ophthalmic campaign, criticised for ‘[l]acking in co-operative spirit with his colleagues’, and ‘Abla, in charge of the syphilis campaign, described as ‘not very interested in this branch of work’.^44^ While they continued to tour the villages of the sub-district across the first years of the revolt, by the second half of 1938 both campaigns stumbled; the village clinics had to be discontinued ‘owing to the increasing lack of security’ (ARDOH 1938: 62). At this stage, even the mechanisms for the notification of births and deaths in the villages around Hebron were breaking down, underlining the scale of the retreat of the mandate government in the countryside (ARDOH 1938: 17).

Medical services were certainly disrupted by the revolt, and medical workers were sometimes the targets of violence and threats. One of Dr Forster’s assistants at St Luke’s, a Syrian doctor by the name of Khoury, received a letter – purportedly from ‘Rebel G.H.Q., Palestine’ – threatening him with death if he did not leave the country within a week; Forster felt it might be a hoax, but Khoury took it more seriously, and left the hospital the following day.^45^ Though the department of health laid the blame for these disruptions squarely at the feet of the rebels, the colonial state had itself, as part of a broader strategy to undermine popular support for the rebels (Kelly 2017: 142), helped create the conditions for chaos and criminality. As Charles Anderson (2017: 47) argues, this was not only through the state’s retreat from the administration of ordinary criminal justice, but even, on occasion, by encouraging brigands and others to impersonate rebels. Medical services, like many other aspects of Palestinian everyday life, were collateral damage in this drive to fracture the cohesion of the revolt.

But a second element in the British counterinsurgency strategy impacted perhaps more profoundly, if still indirectly, on both provision of and access to medical services during the revolt: the use of collective punishment. The principle of collective punishment had been enshrined in law by the British mandate more than a decade before the outbreak of the great revolt, sanctified by an understanding of Palestinian village life as oriented towards mutual protection rather than justice (Hughes 2009: 317). This legal framework was expanded over the course of the great revolt, guiding British counterinsurgents as they demolished Palestinian property, imposed heavy collective fines, demanded forced labour, and installed punitive village occupations between 1936 and 1939. Recent work on the suppression of the great revolt has shifted attention away from instances of particular brutality towards the biopolitical targeting of the conditions of everyday existence for the Palestinian population as a whole (Anderson 2019), and shown how both the revolt and the world war which immediately followed pushed the mandate to reach more deeply into the lives of subjects than ever before, calculating ‘basic needs’ and measuring out the calories needed to stave off the threat of hunger and ensure bare life (Seikaly 2016: 77–102). A close reading of Forster’s diary extends this analysis, by highlighting how the ability of St Luke’s – the only hospital in Hebron and the surrounding area – to provide medical care for the sick and wounded was profoundly disrupted by the British adoption of a counterinsurgency strategy of collective punishment.

While the hospital itself, as we have seen, was unevenly protected from direct intrusions by police and military personnel across the revolt, in at least three ways British counterinsurgency methods constrained its workings. In the first place, the periodic imposition of curfews put a severe strain on the hospital. While Forster was exempt, these curfews – which often lasted a number of days – meant it was difficult and dangerous for both patients and orderlies to access the hospital without being ferried by Forster himself; they also caused supply problems for the hospital, which again Forster had to resolve himself. Following a frustrating meeting with the local military commander who had imposed one such curfew in October 1938, Forster fumed in his diary that the question of hospital supplies had clearly not occurred to this officer. It was, he wrote, ‘quite a new idea to him’; he must have ‘thought a 48-hour fast for sick people was nothing’.^46^ But for patients and their families, the implications of the curfew in terms of access were stark. Forster recounts the chaos which engulfed the hospital one September’s day, when an indefinite curfew was announced for later that same morning:

‘There followed a hectic hour… Each of the many patients of course demanded immediate treatment before rushing home for the curfew. As it was visiting day, there was another section that wished to see their relatives at once, as the midday visiting time was quite impracticable.’^47^

Here, counterinsurgency measures very clearly intruded on the space of the hospital itself, wrenching routine and dislocating patients’ and families’ experiences.

Second, the introduction of new military road regulations from November 1938 which forbade all drivers and passengers from travelling on the roads unless they had a military pass with a photograph impeded Forster’s ability to visit surrounding villages where he conducted weekly out-patient clinics. Again, while Forster himself was able to travel, a general rebel order forbidding Palestinians from taking out these passes meant that those Palestinians with whom he worked did not dare apply for them. Forster could not run the village clinics single-handedly, and so had to temporarily give them up, leaving villagers without accessible medical services. These difficulties were compounded by the closure of key roads with enormous road blocks by the army.^48^ Finally, although the hospital itself was exempt both from the punitive searches and demolitions which saw Palestinian homes ransacked and destroyed, it was not altogether unaffected by these. In September 1939, for instance, a number of houses in Hebron were demolished using explosives after an army patrol was hit by a rebel landmine. One of the houses was just below the hospital. ‘Although we opened every possible window, at least a hundred panes of glass were broken,’ Forster noted, ‘and the poor old hospital clock, “the best time-keeper in Hebron”, fell on its face from a height, and was picked up insensible’.^49^ In all these cases, though the hospital was not the direct or explicit target of collective punishment measures, it was affected in ways which were consistent with the overall punitive purpose of these measures, as degrading the conditions of life for the Palestinian population as a whole in order to render continued rebellion unsustainable (Anderson 2019).

Faced with the degradation of medical services as a result of the wider British counterinsurgency, Palestinian rebels developed their own response in order to ensure medical attention for the wounded. Forster’s diaries, of course, make clear that he treated wounded Palestinian rebels in 1938 and 1939, and some Palestinian doctors – like the grandfather of the historian Sonia Nimr, Dr Sa’id Nimr, a doctor in Jenin in the 1930s (Nimr 2007: 85) – also extended medical care to rebels on an ad hoc, furtive basis across the revolt. But these arrangements do not seem to have been enough on their own to have compensated for the wider drop in the number of admissions to government hospitals, which was evident as early as 1936 (ARDOH 1936, 12). This decline was understood as driven by the not-unjustified fear among wounded Palestinians that entering a government hospital with a bullet wound, for instance, would draw unwanted police attention – or worse, as we have seen. The decline in hospital admissions left British authorities with the question of how exactly, if at all, wounded Palestinian rebels were being treated, and here – alongside the ad hoc care extended to them by government and mission doctors alike – there were reports of more systematic organising around health among rebels. One Anglican clergyman in Jerusalem recounted Palestinian boasts ‘of hospitals in caves’, and ‘of young Arab women being offered good pay to act as nurses’.^50^ Forster heard something similar in Hebron, recording in his diary in October 1938 that ‘wounded rebels have been treated in the hills by their own doctors, of whom, I am told, there are not a few throughout the country’^51^; the next month he had indirect dealings with one of those rebel doctors, apparently a German permanently attached to one of the rebel leaders in the Hebron area.^52^ Against this backdrop, what might otherwise have appeared to be random looting of government property in ‘the thefts of medical supplies from government depots, police stations, and medical kits from various [doctors] in Jerusalem’^53^ took on a different meaning to some contemporary observers, as the supply lines which served rebel medical services.

This assembling of alternative structures of healthcare might well be understood as one strand within the wider attempt by Palestinian rebels to create what the anthropologist Ted Swedenburg (2003: 133–136) has called ‘counterstate apparatuses’. Swedenburg and others (Anderson 2017; Kahba 2011) have focussed in particular on the establishment of rebel courts, in favour of which Palestinian villagers deserted British colonial courts en masse. It appears that these courts were also put to work in ensuring that the poorest Palestinians had access to medical services. In August 1938, as we saw, Dr ‘Abd el-‘Al and his colleague, Dr ‘Abla, were held up on the road out of Hebron and taken to a rebel court in the hills, where they were tried for misdemeanours which included ‘taking too much money from poor patients’.^54^ This was part of a wider pattern, Forster noted, in which those suspected of ‘oppressing the poor, refusing money to the rebels, giving information to the government and the like’ were kidnapped and put on trial in rebel courts in the Hebron area across 1938.^55^

Just as individual medical workers in Hebron took up a range of positions in relation to the mandate government and Palestinian rebels, so too was healthcare more widely implicated in the politics of the great revolt. The health department blamed disruption to access to medical provision on the conditions of insecurity which plagued the countryside as a result of the revolt, but it is clear that British counterinsurgencies strategies – though never identified as a motor of those conditions of disturbance – also played a role in degrading healthcare. To put it another way, the conditions of healthcare at St Luke’s hospital – the periodic panicked rushes for diagnosis, treatment, family visits; the uncertainty and anxiety around supplies, around travel, around access; the injuries which the hospital had to tend to, and the damage which its own physical fabric experienced – had a political aetiology (Hamdy 2008: 554), tied above all to the British pursuit of a strategy of collective punishment. Although the effects of this strategy were in line with the wider aim of making continued Palestinian support for the revolt unsustainable, it also had the unintended consequence of pushing rebels to set up their own medical services, just as the British abnegation of responsibility for enforcing ‘ordinary’ criminal law during the revolt served to strengthen the need for the rebel courts. While Palestine, and European colonies more broadly, have long been seen as testing grounds for new methods of policing and counterinsurgency, more recent work has insisted that the great revolt was also a laboratory for evolving new anti-colonial tactics and visions (Anderson 2021; Winder 2021). The incorporation, however uneven and experimental, of healthcare into the strategies of colonial counterinsurgents and anti-colonial rebels alike makes clear that these insights can be extended to the medical history of the great revolt too.

## Conclusion

For a number of historians, the strategies adopted by both British counterinsurgents and anti-colonial rebels in the second half of the 1930s prefigure or anticipate in important ways the strategies which have been deployed in the decades since by the Israeli occupation regime and Palestinians (Anderson 2019; Khalili 2010; Winder 2020). While not the focus of this article, similar connections might be traced for the medical history of the great revolt. The British incorporation of healthcare into a counterinsurgency strategy of collective punishment may have been uneven and uncalibrated, especially when set alongside the control of access to medical care as a ‘tactic of war’ in the occupied West Bank today (Giacaman et al. 2009; Pfingst and Rosengarten 2012; Puar 2017; Sousa and Hagopian 2011), but it nonetheless might be taken to represent, in embryonic form, the colonial roots of contemporary Israeli practices. In their effort to ensure access to medical services amongst the poorest, meanwhile, the rebels of 1930s Palestine seem to share the priorities of the popular health movement which emerged in the occupied Palestinian territories in the 1970s, and which sought to meet the health needs of the people through the creation of a health infrastructure of resistance, often in defiance of the Israeli permit regime (Barghouti and Giacaman 1990; Wick 2008).

While delineating such genealogies is possible, the ubiquity of these patterns should give pause for thought. Medical anthropologists have made clear that the deliberate degradation of medical provision and access to healthcare as a form of collective punishment can be found in many contexts in the contemporary world (e.g. Varma 2020: 80); the possibilities and perils which face medical workers as they negotiate questions of medical neutrality in times of crisis, meanwhile, are equally widespread (Aciksoz 2016; Hamdy and Bayoumi 2016; Redfield 2013). Rather than treat Palestine as exceptional, then, a medical historical perspective on the great revolt suggests that it was one site at which these wider dynamics played out. Indeed, parallels are evident elsewhere across the Middle East and North Africa in the interwar years, whether in the British recognition in Iraq that political and medical authority might be welded together for colonial advantage (Dewachi 2017: 49); the contested nature of medical neutrality, with Red Cross medical workers and facilities targeted by Italian bombs in Ethiopia (Rubenstein 2021: 36–37); or the conjoined nature of mandatory experiments in evolving new strategies of colonial counterinsurgency and public health management in French Syria (Neep 2012: 131–164). All these cases, including the case of Palestine, underline the broader refusal on the part of European states and the League of Nations to extend international legal norms protecting medical workers in times of war to conflicts between European colonial powers and non-European peoples.

If medical neutrality was far from normative in these interwar colonial contexts, appeals to it nonetheless deserve attention; as Adia Benton and Sa’ed Atshan (2016: 158) conclude, it is precisely in recognising that medical neutrality cannot be taken for granted that we can come to understand medical neutrality ‘as its own potent political stance’. This article has argued that Arab doctors, medical missionaries, British counterinsurgents, and Palestinian rebels were all actively engaged—albeit unequally—in negotiating the legitimate place of medical workers and healthcare during conflict. While colonial counterinsurgents and some doctors understood healthcare and medical authority as means to preserve the status quo, for medical missionaries like Forster and—more particularly—those Palestinian rebels who participated actively in the instantiation of international legal norms and protections on the ground in Hebron, medicine might be put to the service of more radical ends: not simply counterstate formation, but the erasure of those ‘perceived frontiers of civilization’ (Redfield 2016: 263) which the British, like other European powers, cited to avoid recognising these struggles between anti-colonial rebels and colonial counterinsurgents as being a form of war, governed by rules, at all.

## Footnotes

1 Dr A. ‘Abd el-‘Al, Medical Officer Hebron, to Senior Medical Officer, Jerusalem, 8 May 1929, Israel State Archives [hereafter ISA] M[andate Series] 6552/8.

2 Annual Confidential Report for Dr A. ‘Abd el-‘Al, 1930, ISA M 5131/16.

3 Annual Confidential Report for Dr A. ‘Abd el-‘Al, 1931, ISA M 5131/20.

4 Indeed, his only daughter – Marsina – would go on to be engaged to the son of a British military officer in the early 1940s, a connection suggestive of the degree to which he and his family were socially integrated into the British establishment in Palestine. It is important to note, however, that many employees of the Palestine Department of Health had been born outside what became mandate Palestine: many were Syrian, Lebanese, Armenian, and Egyptian, as well as Palestinian. 

5 District Commissioner, Jerusalem, to Chief Secretary, Jerusalem, 26 February 1935, ISA M 205/41.

6 District Commissioner, Jerusalem, to Chief Secretary, Jerusalem 14 September 1936, ISA M 205/41.

7 Annual Confidential Report for Dr A. ‘Abd el-‘Al, 1936, ISA M 5131/12.

8 Annual Confidential Report for Dr A. ‘Abd el-‘Al, 1938, ISA M 5131/12.

9 Diary, 31 August 1938, Elliot Forster Collection, GB165-0109, Middle East Centre Archive [MECA], Oxford. Hereafter “Forster Diary”. He was, however, also treated courteously by the rebel leader, who fed both doctors and sent ‘Abd el-‘Al home with two chickens as a present for his wife.

10 Telegram from Ishaq Abu Khalaf, Hebron, to High Commissioner, Jerusalem, 28 August 1941, ISA M 205/41.

11 District Commissioner, Jerusalem, to Chief Secretary, 6 March 1943, ISA M 205/41.

12 Dr A. ‘Abd el-‘Al, Nazareth, to Director of Medical Services, Jerusalem, 1 November 1946, ISA M 6589/9.

13 Archdeacon, Jerusalem, to Canon Gould, 17 July 1936, MECA GB165-0161 Box 59 File 2.

14 Elliot Forster, Report on St Luke’s Hospital, Hebron, April 1938, MECA GB165-0161 Box 59 File 2.

15 See for examples *al-Difa’,* 9 February 1938, p.1; *al-Difa’,* 27 April 1938, p.1; *al-Difa’,* 17 June 1938, p.1.

16 17 October 1938, Forster Diary.

17 27 September 1938, Forster Diary.

18 Bishop in Jerusalem to District Commissioner, Jerusalem, 20 July 1938, MECA GB165-0161 Box 59 File 2.

19 Elliot Forster, ‘Personal impressions of the night of Friday 19th August 1938 and the morning of Saturday 20th August 1938’, 27 August 1938, MECA GB165-0109.

20 Report by Mr. A. Lees, Temporary Assistant District Commissioner, Hebron, on the events of Friday night, the 19th August, and Saturday morning, the 20th August, 1938, 23 August 1938, MECA GB165-0109.

21 *al-Sirat,* 22 August 1938, p.2 (‘Telephone message from Hebron’).

22 15 October 1938, Forster Diary.

23 15 October 1938, Forster Diary.

24 S.O. Richardson, Jaffa, to Attorney General, Jerusalem, 27 December 1940, ISA M 711/12.

25 15 October 1938, Forster Diary.

26 30 October 1938, Forster Diary.

27 2 December 1938, Forster Diary.

28 7 April 1939, Forster Diary.

29 27 June 1939, Forster Diary.

30 Elliot Forster to Assistant District Commissioner, Hebron, 1 July 1939, MECA GB165-0109.

31 29 August 1939, Forster Diary.

32 27 June 1939, Forster Diary.

33 7 April 1939, Forster Diary.

34 Elliot Forster to Assistant District Commissioner, Hebron, 1 July 1939, MECA GB165-0109.

35 27 June 1939, Forster Diary.

36 Farid Haddad, Inspector of Pharmacies, to Director of Health, Jerusalem, 4 April 1924, ISA M 6552/8.

37 Director of Health, Jerusalem, to Senior Medical Officer, Jerusalem, 2 January 1932, ISA M 6552/8.

38 2 December 1938, Forster Diary.

39 4 January 1939, Forster Diary.

40 Report of the Committee on Development and Welfare Services (1940), Israel State Archives [ISA] P 4187/10, p.61.

41 [n.d.] October 1936, Forster Diary; Medical Officer, Government Hospital Gaza, to Senior Medical Officer, Jaffa, 27 July 1938, ISA M 6599/3.

42 10 October 1938, Forster Diary.

43 12 April 1939, Forster Diary.

44 As per their respective annual confidential reports for 1938, in ISA M 5131/12.

45 11 October 1938, Forster Diary.

46 17 October 1938, Forster Diary.

47 4 September 1939, Forster Diary.

48 1 November 1938, Forster Diary.

49 6 September 1939, Forster Diary.

50 Canon C.T. Bridgeman, Jerusalem, to Bishop in Jerusalem, 29 August 1938, MECA JEM 61/3.

51 15 October 1938, Forster Diary.

52 1 November 1938, Forster Diary.

53 Canon C.T. Bridgeman, Jerusalem, to Bishop in Jerusalem, 29 August 1938, MECA JEM 61/3.

54 31 August 1938, Forster Diary.

55 6 September 1938, Forster Diary.
